# Expected Improvements in the Quantitative Remote Sensing of Optically Complex Waters with the Use of an Optically Fast Hyperspectral Spectrometer—A Modeling Study

**DOI:** 10.3390/s150306152

**Published:** 2015-03-13

**Authors:** Wesley J. Moses, Jeffrey H. Bowles, Michael R. Corson

**Affiliations:** Remote Sensing Division, Naval Research Laboratory, 4555 Overlook Ave. SW, Washington, DC 20375, USA; E-Mails: jeffrey.bowles@nrl.navy.mil (J.H.B.); mike.corson@nrl.navy.mil (M.R.C.)

**Keywords:** F-number, Dyson, HICO, sensor noise, atmospheric correction, coastal waters, water quality, signal-to-noise ratio (SNR), hyperspectral, remote sensing

## Abstract

Using simulated data, we investigated the effect of noise in a spaceborne hyperspectral sensor on the accuracy of the atmospheric correction of at-sensor radiances and the consequent uncertainties in retrieved water quality parameters. Specifically, we investigated the improvement expected as the F-number of the sensor is changed from 3.5, which is the smallest among existing operational spaceborne hyperspectral sensors, to 1.0, which is foreseeable in the near future. With the change in F-number, the uncertainties in the atmospherically corrected reflectance decreased by more than 90% across the visible-near-infrared spectrum, the number of pixels with negative reflectance (caused by over-correction) decreased to almost one-third, and the uncertainties in the retrieved water quality parameters decreased by more than 50% and up to 92%. The analysis was based on the sensor model of the Hyperspectral Imager for the Coastal Ocean (HICO) but using a 30-m spatial resolution instead of HICO’s 96 m. Atmospheric correction was performed using Tafkaa. Water quality parameters were retrieved using a numerical method and a semi-analytical algorithm. The results emphasize the effect of sensor noise on water quality parameter retrieval and the need for sensors with high Signal-to-Noise Ratio for quantitative remote sensing of optically complex waters.

## 1. Introduction

Quantitative remote sensing of inland, estuarine, and coastal waters is inherently challenging due to the optical complexity often encountered in these waters. These waters contain significant concentrations of Suspended Particulate Matter (SPM) and Colored Dissolved Organic Matter (CDOM) in addition to chlorophyll-*a* (chl-*a*) and are, therefore, more optically complex than open ocean waters [1]. For open ocean waters, ±70% is the generally accepted minimum threshold for the required accuracy in estimating chl-*a* concentration [2]. The global research community has not arrived at a similar figure for the required accuracy in retrieved water quality parameters for inland, estuarine, and coastal waters, though it is generally acknowledged that accurate estimation of water quality parameters in inland, estuarine, and coastal waters using remotely sensed data is more challenging because of the complex optical properties of these waters. The high bio-optical variability and spatial heterogeneity of these waters necessitate sensors of higher spectral and spatial resolutions than those offered by standard ocean color sensors (e.g., [3,4,5,6]), such as SeaWiFS (Sea-viewing Wide Field-of-view Sensor), MODIS (MODerate resolution Imaging Spectroradiometer), and MERIS (MEdium Resolution Imaging Spectrometer). Following the launch of the first spaceborne hyperspectral sensor, Hyperion, by NASA (National Aeronautics and Space Administration) in 2000, several spaceborne hyperspectral sensors, such as CHRIS (Compact High Resolution Imaging Spectrometer) and HICO (Hyperspectral Imager for the Coastal Ocean) have been launched and several others are currently under development. NASA has at least three hyperspectral missions with coastal water applications, namely, PACE (Pre-Aerosol, Clouds, and ocean Ecosystem), GEO-CAPE (GEOstationary Coastal and Air Pollution Events), and HyspIRI (Hyperspectral Infrared Imager), which are under various stages of planning and development, with the specific sensor configurations yet to be finalized. As demands grow for sensors with higher spatial and spectral resolutions for coastal water analysis, quantitative analysis of the inherent effect of sensor noise on the accuracy of retrieved water quality parameters would provide valuable information for sensor developers.

There are at least four primary sources of uncertainty in the values of biophysical parameters estimated from remotely sensed data acquired over a water target: (i) inherent errors in the retrieval algorithm, *i.e.*, uncertainties in the relationship modeled by the bio-optical algorithm between the reflectance from water and the biophysical parameter; (ii) errors in the radiometric or spectral calibration of the sensor; (iii) noise in the sensor; and (iv) errors in the atmospheric correction of the remotely sensed data. The sensor noise is a basic source of error that inevitably affects all quantitative retrievals and thus represents an unavoidable minimum level of error in the retrievals. In other words, even if the sensor calibration, atmospheric correction model, and bio-optical algorithms are all completely error-free, errors in the data due to sensor noise alone will unavoidably lead to errors in the atmospheric correction and, consequently, biophysical retrievals.

In our previous study [7], we analyzed the effect of sensor noise alone on the accuracy of retrieved biophysical parameters, assuming error-free atmospheric correction. The objective of that study was to report the minimum error (due to sensor noise) present in the retrieved results even if the atmospheric effects were completely removed. However, in reality, atmospheric effects are seldom removed completely, and the atmospheric correction itself is affected by sensor noise, which adds to the uncertainties in the biophysical retrievals. In the current study, we have included the impact of sensor noise on atmospheric correction and its consequent effect on uncertainties in the retrievals. In particular, we have investigated the reduction in the overall uncertainty in the estimated water quality parameters, namely, the concentrations of chl-*a*, CDOM, and SPM, as one particular component of the sensor configuration, *i.e.*, the F-number, which is the ratio of the imager focal length to the aperture diameter, is changed to yield a better signal-to-noise ratio (SNR). Increasing the optical throughput by using a lower F-number is a conceptually straightforward method to increase the SNR, though there are other ways of improving the SNR and we do not intend to present the change in F-number as the only or even the best method for achieving higher SNR. The results presented here are not impacted by the source of the improved SNR. The objective of this study was to estimate the expected errors in the retrieval of the aforementioned water quality parameters in coastal waters for a nominal sensor configuration (using HICO as the basic sensor model) and provide a broad picture of the expected improvements in the accuracy of the retrievals as the sensor configuration is changed to improve the SNR, with the hope that the results presented and the inferences made herein will serve as useful sources of information for designing new spaceborne hyperspectral sensors for coastal remote sensing.

## 2. Data and Methods

### 2.1. Reflectance at the Water Surface

The radiative transfer model Ecolight [8] was used to synthetically generate remote sensing reflectances (R_rs_) at the water surface. Ecolight is a fast version of Hydrolight [9,10]. It generates radiometric quantities by solving the azimuthally averaged radiative transfer equation, which is suitable for the purpose of this study as we are interested only in the water-leaving R_rs_ in the nadir-viewing direction, whereas Hydrolight solves the complete radiative transfer equation and generates radiance distribution as a function of depth below water and polar and azimuthal angles, which is computationally intensive. R_rs_ spectra were generated for a wide range of concentrations of chl-*a*, CDOM, and SPM, typically encountered in coastal waters. Only low-to-moderate concentrations of SPM were considered because the atmospheric correction program used in this study assumes zero water-leaving radiance in the near-infrared (NIR) wavelengths used for retrieving aerosol parameters. Fourteen different levels of chl-*a* concentration, seven levels of CDOM concentration, and five levels of SPM concentration were used (Table 1), leading to 490 combinations of constituent concentrations, for each of which an R_rs_ spectrum was generated using Ecolight. The absorption coefficient of CDOM at 440 nm (*a*_CDOM_(440)) was used as a measure of the CDOM concentration. The water was assumed to be optically deep to avoid interferences from a reflective bottom, which could affect the retrievals. All other relevant parameters, such as the sediment type, phase function of chl-*a* and sediment particles, *etc.*, were kept the same for all 490 R_rs_ spectra generated through Ecolight simulations. The R_rs_ spectra were generated at the wavelength locations and spectral resolution of HICO, which collects data in the 350–1080 nm range at a 5.7 nm spectral resolution [11]. Only data within the 400–725 nm range were used for retrieving water quality parameters.

**Table 1:** Concentrations of chl-*a* and SPM, and absorption coefficient of CDOM at 440 nm, for which reflectance spectra were generated using Ecolight.

Chl-*a* (mg m^−3^)	SPM (g m^−3^)	*a*_CDOM_(440) (m^−1^)
1, 5, 10, 15, 20, 25, 30, 35, 40, 45, 50, 60, 70, 80	1, 2, 4, 6, 8	0.1, 0.5, 1, 1.5, 2, 5, 10

### 2.2. Propagation to Top-of-Atmosphere (TOA) Radiance

**Figure 1:**
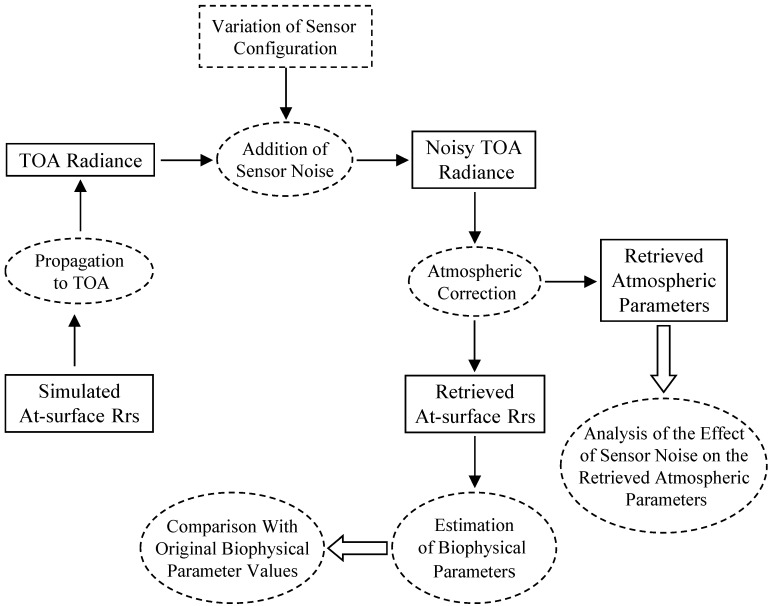
Flow chart of the data processing steps (TOA: top of atmosphere).

The at-surface reflectance data were processed in the sequence shown in Figure 1. The at-surface reflectances were propagated through the atmosphere using the radiative transfer model Tafkaa [12,13] to estimate the TOA (Top Of Atmosphere) radiance at the sensor. Solar illumination and viewing conditions at the sensor, as specified by the date and time of data acquisition, the geographic location of the target, the ground elevation, the sensor altitude, and the viewing angles of the sensor in the zenith and azimuth directions, were kept the same for all 490 at-surface R_rs_ spectra. The location of the target and the date/time used in the simulation resulted in a solar zenith angle of 37 degrees. Atmospheric parameters, such as the atmospheric model, the types of atmospheric gases, the ozone amount, the column water vapor amount, the relative humidity, the aerosol model, the aerosol optical depth at 550 nm, and the wind speed, were also kept the same (Table 2).

**Table 2:** Geographic, illumination, and atmospheric parameter values used for the upward propagation of the at-surface reflectances.

Parameter	Value
Date	9 April 2011
Time	15:30:00 (GMT)
Latitude	37°30′
Longitude	−76°10′
Ground Elevation	0 km
Sensor Altitude	400 km
Sensor Zenith Angle	0°
Sensor Azimuth Angle	0°
Atmospheric Model	Mid-Latitude Summer
Atmospheric Gases	H_2_O, CO_2_, O_2_, N_2_O, CO, CH_4_, O_3_
Ozone Amount	0.34 atm-cm
Column Water Vapor	2.50 cm
Relative Humidity	50%
Aerosol Model	Maritime
Aerosol Optical Depth at 550 nm	0.225581
Wind Speed	2 m s^−1^

### 2.3. Addition of Sensor Noise

Each of the 490 TOA radiance spectra was replicated to generate a 1000-pixel image to which noise was added (see also [7]), resulting in 490 noisy images.

#### 2.3.1. Considerations for the Sensor Configuration

Damm *et al.* [14] studied the effects of SNR, spectral resolution, spectral sampling interval, and spectral shift on the accuracy of the retrieved chl-*a* fluorescence in plants, and concluded that among these four factors the SNR had the greatest effect on the accuracy of the retrieved chl-*a* fluorescence. The configuration of a sensor determines the maximum achievable SNR. While it is desirable to design sensors that yield the highest possible SNR, the final design of a spaceborne sensor is a trade-off amongst various factors, such as the instrument cost, performance requirements (including spectral and spatial resolutions, spectral sampling interval, and dynamic range), and payload constraints.

HICO currently acquires data at a 96-m spatial resolution [11]. Considering the continual push for sensors with higher spatial resolution for detailed coastal studies, it was decided to simulate HICO data at a 30-m spatial resolution for this study. Several hyperspectral sensors scheduled to be launched in the near future, such as HISUI (Hyperspectral Imager Suite), EnMAP (Environmental Mapping and Analysis Program), and PRISMA (PRecursore IperSpettrale della Missione Applicativa—*i.e.*, Hyperspectral Precursor of the Application Mission), are designed to have a spatial resolution of 30 m. For the data simulation purposes in this study, the change in spatial resolution was implemented by keeping the detector pixel size at its current value of 16 μm and increasing the focal length of the lens from 67 to 213.3 mm. HICO has an Offner spectrometer with an F-number of 3.5 [11]. The aperture diameter (= focal length/F-number) was therefore set at 0.061 m.

While there are various ways to modify the sensor configuration and get the desired improvement in SNR, one of the most obvious and straightforward ways is by enlarging the diameter of the aperture, which increases the number of photons incident on the focal plane and improves the SNR across all spectral channels. In this study, we have investigated the improvement in the water quality parameter retrieval as the aperture size is increased to achieve an F-number of 1.0, which is currently the approximate limit of optical spectrometer design and fabrication capability. The Dyson spectrometer [15,16] design allows for an optically fast system with an F-number as small as 1.0. The Portable Remote Imaging Spectrometer (PRISM), built by the Jet Propulsion Laboratory [17], has a Dyson spectrometer with an F-number of 1.8, operating in the 350–1050 nm range, and has been successfully deployed to collect high quality airborne data [18]. In spite of the engineering challenges involved, it is not unreasonable to anticipate the fabrication of a high-fidelity f/1.0 system in the near future.

We do not suggest that using a high-throughput, optically fast spectrometer is the only or the most preferred way of obtaining a high SNR. As alluded to earlier, there are other ways of achieving a higher SNR for the same spatial and spectral resolutions, each of which place different requirements on the sensor design. For e.g., the SNR can be increased through increasing the exposure time by nodding the sensor over the target of interest (through forward motion compensation) as the satellite moves along its orbit. The SNR can also be increased by using larger detector pixels, which would result in an increase in the number of photons captured. This approach would require a longer focal length and, therefore, a larger aperture to maintain the F-number. Nevertheless, there are tradeoffs involved in each of these options. Nodding the sensor reduces the spatial extent of the area that can be captured by the sensor in one orbit. It is not suited for sensors designed to acquire data continuously but for sensors designed to acquire data in an on-demand basis in which high-priority targets are pre-selected and the sensor is programmed to nod when passing over those targets. Moreover, when the sensor nods over a target, the atmospheric path of light from the target to the sensor changes as the satellite moves along. This can cause significant uncertainties in the atmospheric correction, especially in coastal regions adjoining urban areas where the atmospheric aerosol composition may vary significantly over short spatial scales. Using large detector pixels will increase the size and weight of the payload, which can make the mission too expensive and/or difficult to launch. Factors such as the allocated budget for the mission, the allocated payload space in the launch vehicle, the type of orbit, and the intended application of the data drive the decision on the particular option or a combination of options for obtaining optimal SNR.

In this study, we have opted to model the increase in SNR by decreasing the F-number. Keeping the focal length at 213.3 mm, the aperture diameter was increased to 0.2133 m to get an F-number of 1.0. We simulated data for a system with an F-number of 1.0 and compared the results to the results obtained from a HICO-like system with an F-number of 3.5. As there are a number of factors besides the F-number that affect SNR and an increase in SNR can be achieved by adjusting any of these factors, we prefer to cast this study as a comparison between a high-SNR and a low-SNR system rather than strictly as f/3.5 *vs.* f/1.0, while underscoring that in this study the modeled high-SNR was achieved by decreasing the F-number. Henceforth, the low-SNR system refers to a HICO-like f/3.5 system, whereas the high-SNR system refers to an f/1.0 system.

#### 2.3.2. Sensor Noise Model

The noise was added to the signal as a normally distributed random variable with a mean of zero and a standard deviation equal to the expected noise level. The total sensor noise was calculated by adding in quadrature the shot noise, dark noise, readout noise, and digitization noise.

(1) $$Noise=\sqrt{{\left(Shot\;Noise\right)}^{2}+{\left(Dark\;Noise\right)}^{2}+{\left(Readout\;Noise\right)}^{2}+{\left(Digitization\;Noise\right)}^{2}}$$

The shot noise is the square root of the total signal (electrons) generated by the photons incident on the detector. The total signal generated at the detector is given by [19,20],

(2) $$\text{Signal}=\frac{\lambda }{hc}L_{\text{Δ}\lambda }\frac{\pi }{4}\frac{D^{2}}{f^{2}}p^{2}T\eta_{\text{sys}}$$

where, *λ* is the wavelength of the incident radiation (in units of μm)
*h* is the Planck’s constant
*c* is the velocity of electromagnetic radiation (in units of m s^−1^)
$L_{\text{Δ}\lambda }$ is the incoming radiance at the sensor in the waveband Δ*λ* (in units of Wm^−2^ Sr^−1^)
*D* is the diameter of the aperture (in units of m)
*f* is the focal length of the imaging system (in units of m)
*p* is the spatial width of the detector pixel (in units of m)
*T* is the exposure time (in units of s)
$\eta_{\text{sys}}$ is the overall system efficiency, which is given by,
$\eta_{\text{sys}}=\eta_{\text{op}}\times \eta_{\text{QE}}\times \eta_{\text{g}}$ , where,
$\eta_{\text{op}}$ is the optical transmittance of the system
$\eta_{\text{QE}}$ is the quantum efficiency of the detector
$\eta_{\text{g}}$ is the grating efficiency, which is given by,
$\eta_{\text{g}}(\lambda )=\eta_{\text{g}0}{\text{sinc}}^{\text{2}}\left[f_{g}\left(1-\frac{\lambda_{b}}{\lambda }\right)\right]$ , where,
$\eta_{\text{g}0}$ is the grating efficiency at the blaze wavelength, $\lambda_{b}$ ,
sincx=(sinπx)/πx
$f_{g}$ is the fraction of a grating groove that is at the blaze angle.

(3) $$\text{Thus},\text{ the shot noise }=\;\sqrt{\frac{\lambda }{hc}L_{\text{Δ}\lambda }\frac{\pi }{4}\frac{D^{2}}{f^{2}}p^{2}T\eta_{\text{sys}}}$$

*h* (= 6.63 × 10^−34^ Js) and *c* (= 3 × 10^8^ ms^−1^) are constants. Nominal values for the relevant instrument-related quantities in Equation (3) were taken from the corresponding values for HICO. Lucke *et al.* [11] have provided a detailed description of the HICO instrument. For the high-SNR (*i.e.*, F-number = 1.0) system, the quantum efficiency of the commercially available FBX-2K256 CMOS array from Brandywine Photonics (Exton, PA, USA), which has the required electron well depth to remain unsaturated when used in an f/1.0 system over shallow coastal waters, was used in the noise calculation. The quantum efficiency of this detector was slightly higher than that of the HICO detector at wavelengths below 450 nm and above 750 nm and lower than that of the HICO detector at wavelengths between 450 nm and 750 nm.

The standard deviation of a dark image acquired by HICO was taken to represent the combination of dark noise, readout noise, and digitization noise for the low-SNR system. For the high-SNR system, the dark noise, readout noise, and digitization noise were obtained from the information sheet for the FBX-2K256 CMOS detector.

**Figure 2:**
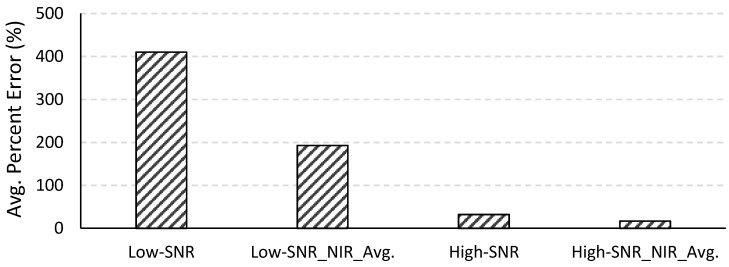
The average SNR calculated from the at-sensor radiances (of all 490 images) for the low- and high-SNR systems. The ratio of the average SNR for the high-SNR system to that for the low-SNR system is plotted on the secondary axis.

In changing the F-number from 3.5 to 1.0 while maintaining the focal length, the diameter of the aperture was increased by 3.5 times, resulting in a 12.25-fold increase in the area of the aperture. Thus, a system with F-number = 1.0 receives 12.25 times more photons than a system with F-number = 3.5, leading to a significant improvement in the SNR. This is illustrated in Figure 2, which contains plots of the average SNR calculated from the at-sensor radiances for all 490 images for both systems. The average SNR for the high-SNR system (F-number = 1.0) is more than four times higher than the average SNR for the low-SNR system (F-number = 3.5) throughout the visible spectral range, where shot noise dominates, and up to 10–12 times higher in the NIR region, where the dark noise and read noise play a dominant role.

#### 2.3.3. Improving Effective SNR through Post-Processing

In addition to making changes to the sensor configuration, the uncertainty in the atmospherically corrected data can be further reduced by post-processing methods. For instance, the noise in the NIR spectral bands used for atmospheric correction can be reduced by averaging the pixels in the spatial or spectral domains. Such averaging, when performed at reasonably moderate scales, will have negligible impact on data analysis because the reflectances at these spectral bands (beyond 750 nm) are often used primarily for atmospheric correction only and not for retrieving water quality parameters. However, spatial averaging is preferred over spectral averaging because spectral averaging, while increasing the SNR, will coarsen the spectral resolution at spectral bands that are used for the retrieval for atmospheric parameters; spatial averaging will not have a detrimental effect on the retrieval of atmospheric parameters if the atmosphere can be safely assumed to be constant over the area represented by the pixels that are averaged. For sensors with high spatial resolution, on the order of a few tens of meters, this is usually a safe assumption, except, perhaps, for waters very close to the coastline. In this study, spatial averaging in the NIR spectral bands was performed by replacing the at-sensor NIR radiance at each pixel with the average of the at-sensor NIR radiances in a 3 × 3 pixel area containing and surrounding the pixel. Considering the cost and complicated engineering effort involved in fabricating an f/1.0 system, it is pertinent to consider whether spatially averaging the data at the NIR spectral bands from an f/3.5 system would yield a comparable reduction in retrieval uncertainty to what would be obtained from an f/1.0 system.

### 2.4. Atmospheric Correction of Noisy TOA Radiance Images

Atmospheric correction is a necessary part of the processing of remotely sensed data, especially if quantitative information (used in conjunction with physical models) is to be retrieved. The simulated noisy at-sensor radiances were atmospherically corrected using Tafkaa to remove the atmospheric effects and retrieve the at-surface reflectances. Tafkaa was set to atmospherically correct the noisy at-sensor radiance images by automatically retrieving the atmospheric parameters from the noisy radiances. Tafkaa uses a look-up-table approach to estimate the atmospheric contribution to the at-sensor radiances. The radiance observed at the sensor is expressed as,

(4) $$L_{\text{obs}}=L_{\text{atm}+\text{sfc}}+L_{\text{w}}t_{u}$$

where *L*_obs_ is the observed radiance at the sensor, *L*_atm+sfc_ is the combination of atmospheric path radiance, the radiance specularly reflected from the water surface, and the radiance from whitecaps that is transmitted through the atmosphere to the sensor, *L*_w_ is the water-leaving radiance, and *t*_u_ is the upward transmittance of *L*_w_ to the sensor. Converting the radiance quantities into reflectance quantities and taking into account the absorption and scattering processes in the ocean-atmosphere system along the sun-surface-sensor path, Equation (4) can be rewritten as (see [13] for details),

(5) $$\rho_{\text{obs}}=T_{g}\left(\rho_{\text{atm}+\text{sfc}}+\frac{\rho_{\text{w}}t_{u}t_{d}}{1-s\rho_{\text{w}}}\right)$$

where $\rho_{\text{obs}}$ is the observed at-sensor reflectance, $\rho_{\text{atm}+\text{sfc}}$ is the at-sensor reflectance due to the combination of atmospheric path radiance, specular reflection from the water surface, and reflection from whitecaps, $\rho_{\text{w}}$ is the apparent water-leaving reflectance, $t_{d}$ is the downward atmospheric transmittance of the solar irradiance, *s* is the average reflectivity of the atmosphere for isotropic radiation upwardly incident at its base, and *T_g_* is total atmospheric gaseous transmittance in the sun-surface-sensor path. The term $1-s\rho_{\text{w}}$ accounts for the loss of photons due to the downward reflection of a part of the water-leaving radiance by the atmosphere back to the water surface. The effects of subsequent multiple reflections of photons between the water surface and the atmosphere are neglected. Rearranging terms,

(6) $$\rho_{\text{w}}=\frac{\rho_{\text{obs}}/T_{g}-\rho_{\text{atm}+\text{sfc}}}{t_{u}t_{d}+s\left(\rho_{\text{obs}}/{\text{T}}_{\text{g}}-\rho_{\text{atm}+\text{sfc}}\right)}$$

Thus, $\rho_{w}$ (from which R_rs_ is calculated by division by π) can be determined if the quantities on the right-hand-side (RHS) of Equation (6) are determined through measurements or theoretical modeling. Tafkaa uses pre-computed values (stored in a look-up-table) of the quantities on the RHS of Equation (6), calculated using a slightly modified version of the vector radiative transfer model developed by Ahmad and Fraser [21] for a variety of solar illumination and sensor viewing geometries and five aerosol models (each at five levels of relative humidity and 10 levels of optical thickness). For illumination/viewing geometries and atmospheric conditions not explicitly contained in the look-up-table, Tafkaa interpolates using adjacent values to determine the radiometric quantities.

One of the goals of this study was to analyze the effect of sensor noise on atmospheric correction by comparing two retrieved atmospheric parameters, namely, the column water vapor amount and the aerosol optical thickness, with the original atmospheric parameters that were used to propagate the at-surface reflectances to TOA radiances. To that end, atmospheric correction was performed by setting the column water vapor amount, aerosol optical thickness, relative humidity, and aerosol model as variable parameters and fixing the rest of the parameters at the same values (Table 2) that were used to propagate the original at-surface reflectances to TOA radiances. Radiances in the NIR spectral channels closest to 750 nm, 865 nm, and 1040 nm were used to retrieve the aerosol properties.

### 2.5. Estimation of Water Quality Parameters

Two approaches were used to estimate the water quality parameters from the atmospherically corrected data: (i) a non-linear least squares error minimization approach to estimate the concentrations of chl-*a* and SPM and *a*_CDOM_(440) simultaneously and (ii) a semi-analytical NIR-red algorithm to estimate chl-*a* concentration.

(i) Non-Linear Least Squares Error Minimization Approach:

The concentrations of chl-*a* and SPM and *a*_CDOM_(440) were estimated from the atmospherically corrected noisy at-surface reflectances by using the Levenberg-Marquardt method [22,23], which is a non-linear least squares curve fitting procedure that estimates parameters by minimizing the squared difference between measured data and modeled data. In this case, the estimated parameters were the concentrations of chl-*a* and SPM and *a*_CDOM_(440); the atmospherically corrected noisy at-surface reflectances were treated as the measured data; and the modeled data were obtained from fresh runs of the radiative transfer model Ecolight for a given set of parameters. The parameter estimation was done through MPFIT [24], which is an enhancement of the FORTRAN-based software MINPACK [25,26], and is written for use with IDL (Interactive Data Language). For a given noisy spectrum, MPFIT takes in an initial set of parameter values, which were set equal to the corresponding original constituent concentrations (Table 1), and through an iterative process estimates the optimal concentrations of chl-*a* and SPM and *a*_CDOM_(440) by minimizing the squared difference between the noisy R_rs_ spectrum and the R_rs_ spectrum generated by Ecolight for a given set of parameters.

The numerical approach for estimating the water quality parameters was computationally intensive and time consuming, and was therefore not applied to all 490 images. Instead, 20 images encompassing ranges of constituent concentrations (Table 3) typically encountered in coastal waters were selected and used for this analysis. These 20 images constitute a representative sample that spans the entire range of chl-*a* and SPM concentrations encompassed by the 490 images. Very high *a*_CDOM_(440) values were omitted from this subset because the spectral overlap of the absorption features of chl-*a* and CDOM in the blue spectral region introduces additional retrieval errors in algorithms that use reflectances in the blue region. The omission of very high *a*_CDOM_(440) values was considered acceptable because this study is not a test of the robustness of the algorithm but rather its sensitivity to uncertainties caused by sensor noise, which meant that the uncertainties due to inherent errors in the bio-optical algorithm had to be kept at a minimum so that the uncertainties observed in the retrievals can be confidently attributed largely to the effects of sensor noise.

**Table 3:** Constituent concentrations considered for the analysis of the effect of sensor noise on the water quality parameters retrieved through the non-linear least squares error minimization approach.

S. No.	Chl-*a* (mg m^−3^)	*a*_CDOM_(440) (m^−1^)	SPM (g m^−3^)
1	1	0.1	1
2	5	0.1	2
3	5	0.5	1
4	10	0.1	4
5	10	1	6
6	15	0.5	4
7	20	0.1	2
8	20	0.5	4
9	25	2	8
10	30	0.1	2
11	30	1	6
12	35	1.5	8
13	40	1	2
14	45	1.5	4
15	50	1	8
16	50	2	4
17	60	1.5	8
18	70	1.5	4
19	70	0.5	6
20	80	2	8

(ii) Semi-Analytical NIR-red Algorithm:

The chl-*a* concentration was also estimated using the semi-analytical two-band NIR-red model [27].

(7) $$\text{Two}-\text{Band NIR}-\text{red Model}:\text{ Chl}-a\propto \frac{R_{\text{NIR}}}{R_{\text{red}}}$$

where R_NIR_ and R_red_ are reflectances in the NIR and red regions, respectively. The two-band NIR-red model has been previously shown to yield accurate estimates of chl-*a* concentration (e.g., [28]) when applied to data from MERIS, which has spectral channels centered at 665 nm (red) and 708 nm (NIR). Because HICO does not have a spectral channel centered at 665 nm, the average of the reflectances at 662 nm and 668 nm were used instead.

The simulated dataset contained a wide range of *a*_CDOM_(440) values at seven discrete levels for each combination of chl-*a* and SPM concentrations. This meant that for a given pair of chl-*a* and SPM concentrations, there were seven different reflectance spectra, each varying based on the *a*_CDOM_(440) value, resulting in seven different values for the two-band NIR-red algorithm, which made it not possible to derive a single robust regression relationship between the two-band NIR-red ratio and chl-*a* concentration for the whole dataset. Therefore, the two-band model was parameterized separately (Table 4) at each level of *a*_CDOM_(440) instead of using a single regression model for the entire dataset. This is acceptable because the goal here is not to develop a universally applicable chl-*a* algorithm but to simply test the sensitivity of the two-band NIR-red model to uncertainties arising from the sensor noise. The regression equations (Table 4) were applied to the original, noiseless R_rs_ spectra at each *a*_CDOM_(440) level to estimate the noiseless, “true” chl-*a* concentration according to the two-band NIR-red algorithm. The same equations were also applied to atmospherically corrected, noisy reflectance data, to estimate the “noisy” chl-*a* concentrations, which were compared with the “true” chl-*a* concentrations to assess the impact of sensor noise on the estimated chl-*a* concentration.

**Table 4:** Coefficients of the second order polynomial regression $\text{Chl}-a=\text{A}x^{2}+\text{B}x+\text{C}$, where $x=R_{708}/\text{avg}.(R_{662},\;R_{668})$, at various levels of *a*_CDOM_(440).

*a*_CDOM_(440) (m^−1^)	A	B	C
0.1	264.16	−200.21	34.499
0.5	271.61	−215.54	39.562
1	279.74	−232.86	45.419
1.5	286.88	−248.64	50.882
2	294.82	−265.68	56.956
5	341.05	−367.56	96.38
10	429.34	−567.56	186.18

### 2.6. Measures of Uncertainty

Uncertainty in the results due to the effects of sensor noise was estimated using two error estimates, namely, the average Percent Normalized Root Mean Square Error (PNRMSE) and the average percent error in each parameter retrieved. The average PNRMSE is an estimate of the average variation of the retrieved values from the true value within each image and is, therefore, a measure of the spread of the noise-induced uncertainties in the retrievals within each 1000-pixel noisy image. The average percent error is an estimate of the average departure of the mean retrieved value (the mean calculated from the 1000 pixels of each image) from the true value. The average percent error could reveal any systematic deviation of the mean retrievals from the true values.

The average PNRMSE for each parameter of interest was calculated from the 490 noisy images as follows:

(8) $$\text{Average PNRMSE }=100\times \frac{1}{490}\sum_{j=1}^{490}\;\left(\frac{\sqrt{\frac{1}{1000}\sum_{n=1}^{1000}{\left(X_{noisy\_(j,n)}-X_{original\_j}\right)}^{2}}}{X_{original\_j}}\right)$$

where, *X* is the parameter of interest, $X_{noisy\_(j,n)}$ is the parameter value for the *n*^th^ pixel in the *j*^th^ noisy atmospherically corrected 1000-pixel image, and $X_{original\_j}$ is the parameter value for the original reflectance spectrum used to generate the *j*^th^ image. The average percent error for each parameter of interest was calculated as follows:

(9) $$\text{Average Percent Error}=100\times \frac{1}{490}\sum_{i=1}^{490}\;\left(\frac{\left|{\bar{X}}_{\text{noisy\_}i}-X_{\text{original\_}i}\right|}{X_{\text{original\_}i}}\right)$$

where, ${\bar{X}}_{\text{noisy\_}i}$ is the average parameter value retrieved from the 1000 pixels of the *i*th atmospherically corrected noisy 1000-pixel image and $X_{\text{original\_}i}$ is the corresponding original parameter value that was used to produce the R_rs_ spectrum from which the noisy image was generated.

## 3. Results and Discussion

### 3.1. Effects of Sensor Noise on Atmospheric Correction

**Figure 3:**
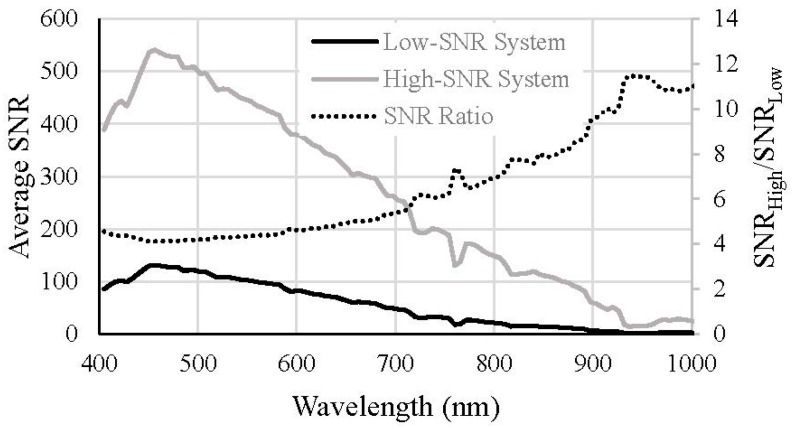
The average PNRMSEs of the atmospherically corrected R_rs_ spectra from the 490 images for the low- and high-SNR systems and the percent improvement (on the secondary axis) in the variations in R_rs_ when the F-number is changed from 3.5 to 1.0.

The effects of sensor noise on atmospheric correction were analyzed by examining the variations in two retrieved atmospheric parameters, namely, the aerosol optical depth at 550 nm (τ_550_) and the atmospheric column water vapor amount (C_wvap_), in addition to the variations in the atmospherically corrected R_rs_ spectra. For the atmospherically corrected R_rs_ spectra, the average percent errors and PNRMSEs (Figure 3) were calculated for each spectral band.

**Figure 4:**
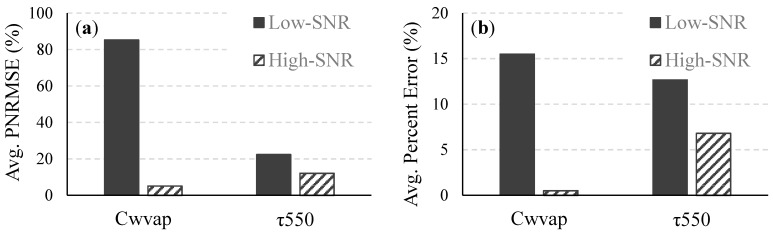
Average (**a**) PNRMSE and (**b**) percent error of C_wvap_ and τ_550_ retrieved from all 490 images for the low- and high-SNR systems.

**Figure 5:**
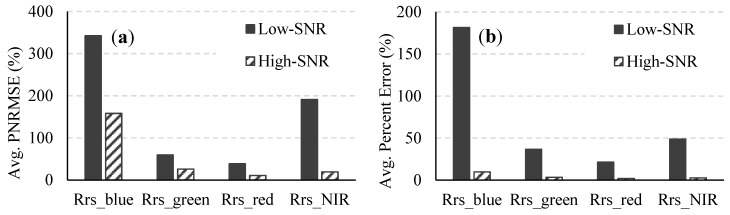
Average (**a**) PNRMSE and (**b**) percent error of the reflectances retrieved from all 490 images for the low- and high-SNR systems.

The SNR in the NIR region had a significant effect on the atmospheric correction, which uses reflectances at NIR wavelengths for estimating column water vapor amount and atmospheric aerosol parameters. For the low-SNR system, the average percent error in the retrieved Cwvap for all 490 images was 15.5% and the average percent error in the retrieved τ550 was 15.1% (Figure 4). The corresponding figures for the high-SNR system were 0.5% and 6.8%, respectively, which translated to 97% and 55% improvements in the retrieved Cwvap and τ550. The average percent error in the retrieved reflectances (Figure 5) improved by 95%, 91%, 91%, and 95% in the blue (400–500 nm), green (500–600 nm), red (600–700 nm), and NIR (700–750 nm) regions, respectively, as the F-number was changed from 3.5 to 1.0. The percent improvement is essentially the percent reduction in the retrieval uncertainty and was calculated as Improvement (%) = $\left(\frac{{\text{[Error]}}_{\text{Low-SNR}}-{\text{[Error]}}_{\text{High-SNR}}}{{\text{[Error]}}_{\text{Low-SNR}}}\right)\times 100$. Similar significant improvements due to the change in F-number were also seen in the variation of the retrieved parameter value within each atmospherically corrected noisy 1000-pixel image. The average PNRMSE of the retrieved C_wvap_ decreased from 85.3% to 5.1%. Significant reductions were also obtained in the variations of the retrieved τ_550_ and reflectances across the whole visible-NIR spectral range (Figure 4 and Figure 5).

**Figure 6:**
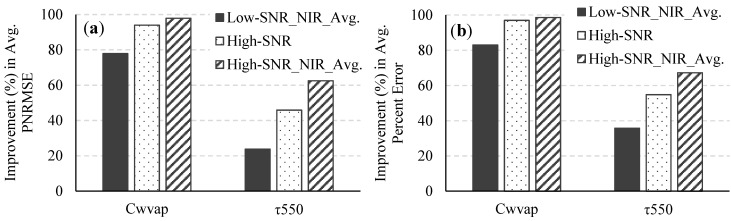
Improvements in (**a**) the average PNRMSE and (**b**) the average percent error of the retrieved C_wvap_ and τ_550_ for the low-SNR system with spatial averaging in the NIR spectral bands, the high-SNR system, and the high-SNR system with spatial averaging in the NIR spectral bands over what was obtained for the low-SNR system.

**Figure 7:**
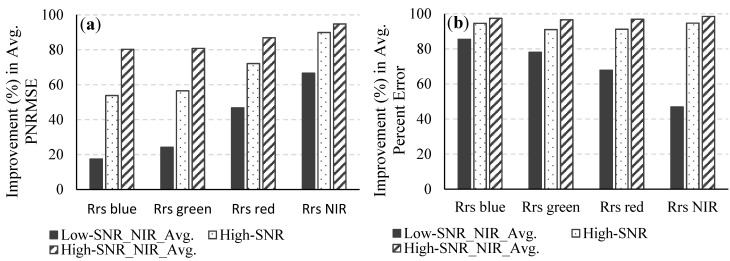
Improvements in (**a**) the average PNRMSE and (**b**) the average percent error of the retrieved R_rs_ for the low-SNR system with spatial averaging in the NIR spectral bands, the high-SNR system, and the high-SNR system with spatial averaging in the NIR spectral bands over what was obtained for the low-SNR system.

Figure 6 and Figure 7 illustrate the percent improvements in the average percent error and the PNRMSEs of the retrieved C_wvap_, τ_550_, and R_rs_ when reflectances in the NIR spectral bands are spatially averaged for both systems; The results show that the retrieval uncertainty for the high-SNR system (without spatial averaging) is significantly lower than the uncertainty for the low-SNR system with spatial averaging at the NIR spectral bands. It must be noted that spatial variations in the atmosphere within the area covered by the pixels that are spatially averaged have been ignored in this study. Therefore, in a real scenario, especially in coastal regions near urban areas, where the atmosphere often changes on a much smaller spatial scale than over the open ocean, the retrieval uncertainty might be higher for the case of spatial averaging at the NIR spectral bands. Averaging over a higher number of pixels, say, 5 × 5, 7 × 7, *etc.* will produce different results, with increased smoothing out of noise but at the risk of removing real spatial variations in the data.

**Figure 8:**
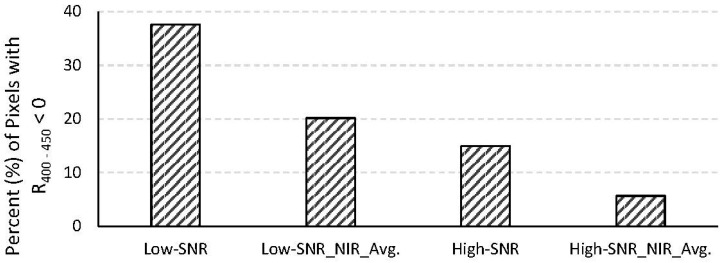
Average percentage of pixels with negative reflectances in the 400–450 nm region for the low- and high-SNR systems with and without spatial averaging in the NIR spectral bands.

Erroneous retrievals of atmospheric parameters during atmospheric correction, especially overestimation of the contribution from atmospheric aerosols to the TOA radiance, can cause over-correction of the at-sensor radiance, resulting in negative reflectances, particularly in the blue region. This is a critical problem, especially in coastal waters near land-water transition zones where there is significant variability in atmospheric aerosols. Overestimation of aerosol contribution can be due to violation of the dark-pixel assumption at the wavelengths used for aerosol retrieval (e.g., [29]) and/or noise in the spectral channels at those wavelengths. In our study, the SPM concentrations were kept very small and the original at-surface reflectances were made to be zero at the NIR spectral bands used for aerosol retrieval. Therefore, errors in the aerosol retrieval can be attributed fully to sensor noise. Negative reflectances in the blue region render the retrieved water quality parameters invalid or highly questionable (depending on the algorithm used for retrieving the water quality parameters). Figure 8 shows the average (from the 490 images) percentage of pixels with negative reflectances in the entire 400–450 nm region for data from the low-SNR and high-SNR systems with and without spatial averaging at the NIR spectral bands. The high-SNR system produces significantly fewer pixels with negative reflectances in the blue region.

### 3.2. Effects of Sensor Noise on the Estimated Water Quality Parameters:

(i) Non-Linear Least Squares Error Minimization Approach:

Concentrations of chl-*a* and SPM and *a*_CDOM_(440) were simultaneously estimated from the 20 (Table 3) atmospherically corrected images. In a few rare cases, the numerical method converged to extremely high, unrealistic values for the water quality parameters, due to noisy spikes in the data. Such pixels were considered invalid and omitted from the error calculations.

**Figure 9:**
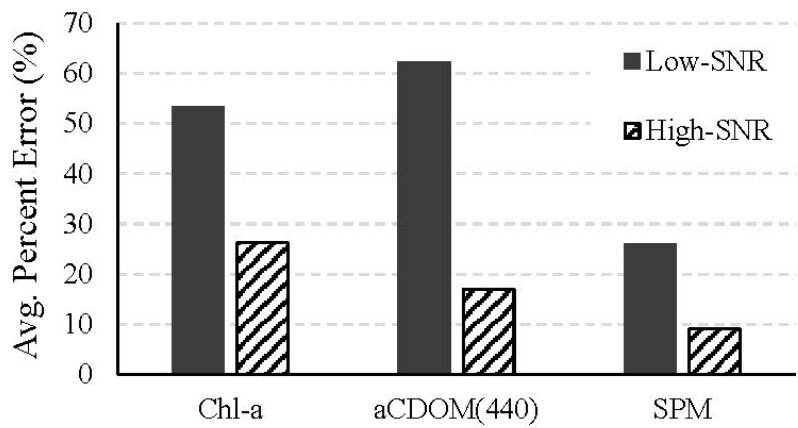
The average percent errors for the low- and high-SNR systems in the concentrations of chl-*a* and SPM and *a*_CDOM_(440) retrieved from the 20 atmospherically corrected noisy images using the non-linear least squares error minimization approach

**Figure 10:**
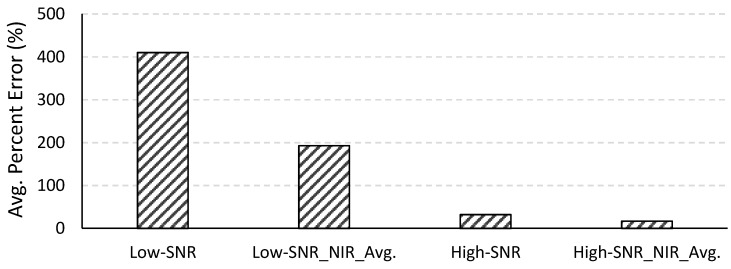
Average percent errors in the chl-*a* concentrations retrieved using the two-band NIR-red algorithm for the low- and high-SNR systems with and without spatial averaging in the NIR spectral bands.

The average percent errors in the retrieved water quality parameters were calculated (Figure 9). On average, the error in the retrievals from the high-SNR system was less than half of that from the low-SNR system for chl-*a* concentration, almost one-fourth for *a*_CDOM_(440), and almost one-third for SPM concentration. The error in chl-*a* concentration for the high-SNR system is within the 30% threshold that is generally accepted as the minimum accuracy required for estimating chl-*a* concentration in open ocean waters [2]. Spatially averaging the NIR reflectances from the low-SNR system resulted in an increase in the number of valid pixels for water quality parameter retrieval (from 68.2% to 82%); however, the retrieval error in the valid pixels was no better than that for the low-SNR system without spatial averaging for chl-*a* and SPM concentrations and better by 35% for *a*_CDOM_(440). When the reflectances from the high-SNR system were spatially averaged at the NIR wavelengths, the retrieval error reduced to 7.01% for chl-*a* concentration, 3.43% for SPM concentration, and 4.88% for *a*_CDOM_(440).

(ii) Semi-Analytical NIR-red Algorithm:

The two-band NIR-red algorithm is not computationally intensive and was therefore applied to all 490 images. Figure 10 shows the average percent errors of the estimated chl-*a* concentrations for the low- and high-SNR systems with and without spatial averaging at NIR wavelengths. The average percent error for the low-SNR system was very high (410.12%). Algorithms such as the two-band NIR-red algorithm that are empirically parameterized are very sensitive to the coefficients of the regression equation governing the algorithm. These algorithms are built on the assumption that variations in the reflectances at the wavelengths included in the algorithm are primarily, if not solely, due to variations in the water quality parameter of interest. As such, these algorithms are very sensitive to variations in the reflectances due to any other factor such as sensor noise. This is important to bear in mind when assessing the NIR-red algorithm because the effects of the sensor noise are expected to be higher in the longer wavelengths. The numerical method, on the other hand, considers the entire spectrum while retrieving water quality parameters (400–725 nm in our case) and can tend to average out the noise across the spectrum without being overly sensitive to noise in one or two particular wavelengths. The absolute error yielded by a semi-analytical algorithm can be mitigated by adjusting the coefficients of the regression equation. The emphasis here is, therefore, not on the absolute error in the chl-*a* concentrations retrieved from the NIR-red algorithm but on the improvement achieved by spatially averaging the NIR reflectances and changing the F-number. When NIR reflectances from the low-SNR system were spatially averaged, the percent error in the retrieved chl-*a* concentration reduced to 192.89%. The percent error for the high-SNR system was 32.06% without NIR spatial averaging and 16.86% with spatial averaging at the NIR spectral bands. In summary, the error in the retrieved chl-*a* concentration reduced by 92.18% when the F-number was changed from 3.5 to 1.0. For both systems, spatial averaging in the NIR wavelengths resulted in approximately 50% reduction in error in the retrieved chl-*a* concentration.

## 4. Conclusions

The results presented here provide a general idea of the expected errors in the atmospheric correction and their consequent effects on the estimated chl-*a* concentration in coastal waters due to sensor noise in a spaceborne hyperspectral sensor with 30 m spatial resolution and 5.7 nm spectral resolution. The results illustrate that uncertainties due to sensor noise in an f/3.5 system with the aforementioned spatial and spectral characteristics, compounded by uncertainties in the atmospheric correction process, are too high to enable accurate quantitative assessment of water quality in optically complex coastal waters. Decreasing the F-number from 3.5 to 1.0 improves the SNR tremendously and leads to more accurate retrievals of atmospheric parameters and, consequently, water quality parameters of water. There are other sensor design considerations for improving the SNR besides decreasing the F-number, which may place less stringent demands on the optical engineering effort. Nevertheless, each such option has its own tradeoffs that need to be evaluated in the context of factors, such as the financial budget and application scope of the mission and the practical logistics of launching the sensor. In this study, we have quantitatively considered the benefit of using an f/1.0 spaceborne hyperspectral sensor for retrieving water quality parameters in Case II waters. In spite of the engineering challenge involved, we believe that recent advances in spectrometer and detector technology make it reasonable to expect an operational spaceborne hyperspectral sensor with an f/1.0 spectrometer in the near future.

Changing the F-number from 3.5 to 1.0 resulted in the following improvements in the retrievals over what was obtained with the f/3.5 system: it reduced uncertainties in the atmospherically corrected reflectance by more than 90% across the visible-NIR spectrum; it reduced the occurrences of invalid, negative reflectances in the atmospherically corrected data to almost one-third; it reduced the uncertainties in the water quality parameters retrieved using the numerical method by approximately 51% for chl-*a* concentration, 73% for *a*_CDOM_(440) and 65% for SPM concentration; it reduced the uncertainty in the chl-*a* concentration by the semi-analytical NIR-red algorithm by 92%.

Apart from changing the sensor configuration, there are other, data-driven ways of improving the SNR, such as averaging the reflectances along spatial or spectral dimensions. However, such measures have inherent uncertainties associated with them, which may or may not be significant depending on factors such as the area of study, the algorithm used to retrieve the biophysical parameters, and the intended application. Such measures can be used to improve the effective SNR for data from a given system, but our results suggest that improvements through such measures alone are not comparable to improvements possible through changing the sensor configuration; neither are they sufficient to reduce the uncertainties in quantitative retrievals of water quality parameters from a spaceborne hyperspectral, high spatial resolution sensor with an f/3.5 optical system to within the desired 30% threshold.

The estimated retrieval errors due to sensor noise, as reported in this study, also include inherent errors in the algorithm used to estimate chl-*a* concentration. The actual error in the estimated chl-*a* concentration will of course ultimately depend on the specific bio-optical algorithm used to retrieve chl-*a* concentration from the reflectance data and will be different for different algorithms. The numerical approach adopted in this study for estimating chl-*a* concentration considers the entire wavelength range between 400 and 725 nm, is not extremely sensitive to noise effects at any particular spectral region, and is therefore suitable for assessing the overall effects of noise on the water quality parameter retrieval. The results show that for a spaceborne hyperspectral sensor with a spatial resolution of 30 m and a spectral resolution of 5.7 nm, using a high throughput Dyson spectrometer with F-number = 1.0 could help estimate chl-*a* concentrations in coastal waters with errors less than the 30% threshold.

It should be noted that the atmospheric correction method employed in this study used reflectances in the NIR region for retrieving atmospheric aerosol properties. The impact of sensor noise on atmospheric correction might be different for a sensor that has spectral channels in the Short Wave Infrared (SWIR) region, in which case, reflectances in the SWIR channels can be used to retrieve atmospheric aerosol properties. When available, SWIR channels are preferred over NIR channels for atmospheric correction (e.g., [30,31]) because the water-leaving radiance is negligible in the SWIR region, even at moderately high concentrations of sediments, due to strong absorption by water and decreased scattering by sediments. The use of SWIR channels will lead to improved retrieval of atmospheric aerosol parameters, especially in cases of turbid waters. However, the decreased signal strength at SWIR channels would require detectors with very high sensitivity at these wavelengths. Further study is required to characterize the effects of noise at SWIR channels and assess the effective improvement in the accuracy of the retrieved water quality parameters due to the use of SWIR channels instead of NIR channels for atmospheric correction.

The interpretations made here of the effect of sensor noise on atmospheric correction were based on the performance of Tafkaa. One might wonder how the inherent limitations and errors in Tafkaa might have contributed to the observed uncertainties in the retrievals that have been attributed to sensor noise in this paper, and whether the outcome and the implications might be different if a different atmospheric routine, say, one based on MODTRAN (MODerate resolution atmospheric TRANsmission) [32], were used instead of Tafkaa. Tafkaa considers all standard atmospheric models, aerosol models, aerosol size parameters, and primary atmospheric gases that are commonly included in standard MODTRAN-based atmospheric correction routines [33]. The look-up-table used in Tafkaa is discretized at intervals that are fine enough to resolve reflectance differences as small as 0.001. Therefore, the results produced by Tafkaa should be comparable to what one might obtain from a MODTRAN-based routine. Moreover, uncertainties due to Tafkaa’s inherent absolute performance errors were largely avoided because the analysis of noise-induced uncertainties was not based on comparison to actual ground truth but based on observed noise-induced variations in the output from Tafkaa (that was generated using input data simulated using the same radiative transfer model with pre-determined atmospheric parameters).

It should be also noted that there are other factors besides sensor noise that affect atmospheric correction and water quality parameter retrieval—factors, such as radiometric calibration, spectral calibration, and the effects of shallow bottom, which have not been considered in this study and would be the subject of a subsequent study in the future.
